# Enzymes as Targets for Drug Development II

**DOI:** 10.3390/ijms24043258

**Published:** 2023-02-07

**Authors:** Sung-Kun Kim

**Affiliations:** Department of Natural Sciences, Northeastern State University, Broken Arrow, OK 74014, USA; kim03@nsuok.edu; Tel.: +1-918-449-6414

Enzymes are viewed as the most desirable targets for drug development by the pharmaceutical community. The accurate characterization of enzymes is essential in comprehending their reactions, and various analytical methods are necessary to achieve this. Purification, kinetics, protein stabilization, the assessment of optimal conditions for pH, temperature, and ionic strength, substrate/product binding, ligand/inhibitor/protein interactions, 3D structure, and conformational changes are some of the key techniques. In recent times, in silico analysis has made a substantial contribution to enzyme characterization, with molecular docking and dynamics being major methods. This Special Issue brings together a diverse range of fascinating topics to inspire thoughts on enzymes as targets for drug development.

Computational methods are becoming increasingly crucial in determining the details of enzyme–inhibitor interactions. The precision of these methods has been significantly enhanced. Molecular dynamics simulations, in particular, play a leading role in providing structural insights through computational approaches. Computational methods are essential in the study of enzyme inhibitors and are heavily relied upon throughout this special topic series of articles. A study conducted by Parise et al. used density functional theory to investigate the inhibition mechanism of the SARS-CoV-2 main protease (Mpro) by ebselen (EBS) and its analog, EBS-OH [1]. The apo form of Mpro was analyzed through molecular dynamics simulations, taking into consideration the nature of His41. The results revealed that EBS-OH demonstrated different behavior compared to EBS in forming a noncovalent complex with Mpro, and the understanding of the inhibition mechanism can support the creation of more effective and selective inhibitors for antiviral therapies.

With computational approaches, kinetic studies are also crucial for comprehending enzyme inhibition. Grodner et al. published three articles. The first article focuses on the examination of two newly patented aminoalkanol derivatives and their effect on prostate acid phosphatase [2]. The inhibition was evaluated through capillary electrophoresis and compared with Lineweaver–Burk plots of the enzyme’s K_m_ with and without the inhibitors. It was found that an aminoalkanol derivative demonstrated strong competitive inhibition compared with another aminoalkanol derivative. This result raises questions about the potential use of these compounds in treating prostate diseases. The second article evaluates the inhibition efficacy of four aminoalkanol derivatives on acetylcholinesterase in vitro [3]. The Lineweaver–Burk plots and K_m_, V_max_, K_i_, and IC_50_ values showed that all four derivatives are competitive inhibitors of acetylcholinesterase and that their inhibitory potency varies based on the substituent present in the main compound structure. The most potent inhibitors contain isopropylamine or methyl substituents, while dimethylamine or ethyl substituents have a weaker effect. Docking studies suggest that the compounds bind with the peripheral anionic site and not the catalytic pocket due to the presence of a sterically extended substituent. The third article investigates the type and strength of catalase inhibition by two pairs of aminoalkanol derivatives [4]. The results indicate they are all competitive inhibitors of catalase, but their potency varies depending on the substituents in their main structure. Capillary electrophoresis was used to monitor changes in substrate and product concentrations and showed that all derivatives are weak inhibitors of catalase, which is an advantage as catalase inhibition leads to an increase in harmful reactive oxygen species. The results of docking studies also confirm their inhibitory strength.

Bodourian et al. and Huckleby et al. conducted research on the other kinetic studies along with an in silico analysis. Bodourian et al. evaluated the ligandability of the human glutathione transferases isoenzyme (hGSTM1-1) using various pesticides as probes [5]. The results showed the limited ligandability and ligand-binding promiscuity of hGSTM1-1 compared to other GSTs and identified pirimicarb as the strongest inhibitor. A crystal structure of hGSTM1-1 was determined, and a comparative analysis showed it interacts preferentially with one-ring aromatic compounds. The study provides a basis for designing new drugs targeting hGSTM1-1. Huckleby et al. studied the issue of antibiotic resistance caused by lactamases, including the dangerous metallo-β-lactamases (MBLs), which differ from serine-β-lactamases [6]. The researchers investigated the potential of two compounds containing two hydroxamate functional groups to inhibit MBL from Bacillus anthracis (Bla2). The results of in silico docking and molecular dynamics simulations showed that the compounds coordinated with zinc ions in the active site of MBLs, and an in vitro kinetic analysis revealed that the mode of inhibition was competitive with effective Ki values for the two compounds. These findings suggest that compounds with dihyroxamate moieties may offer a new way to overcome antibiotic resistance.

In this research topic, the interactions between enzyme and ligand are studied through the use of 3D structures and other aforementioned techniques. Robescu et al. explored the reservoir of filamentous fungi to expand the portfolio of Old Yellow Enzymes (OYEs) as useful biocatalysts [7]. They discovered four new members of the OYE superfamily in the genomes of Aspergillus niger and Botryotinia fuckeliana. BfOYEs showed wider substrate spectra than their AnOYE counterparts, which were more specialized. The crystal structures of BfOYE4 and AnOYE8 were determined and showed unique features, such as a peculiar N-terminal loop, while models of BfOYE1 and AnOYE2 showed surprisingly wide access to the active site cavities. Liu et al. discuss the importance of phosphorylation in biological events and the balance between phosphorylation and dephosphorylation, which is controlled by protein kinases (PKs) and protein phosphatases (PPs), respectively [8]. They focus on one specific protein tyrosine phosphatase (PTP), PTP1B, which plays a key role in human diseases but is difficult to target due to the lack of specificity among PTP inhibitors. The authors summarize three classes of PTP1B inhibitors with different mechanisms, including targeting multiple aryl-phosphorylation sites, allosteric sites, and specific mRNA sequences, and argue that these inhibitors are promising for the development of efficient small-molecule drugs targeting PTP1B. Müller et al. found that PII-family signal-transducing effectors play a critical role in regulating cellular metabolic fluxes, particularly the nitrogen metabolism [9]. The interaction between these effectors and their targets is controlled by cellular nitrogen levels and energy charge. Structural studies on GlnK, a PII-family effector that regulates ammonium transporters, showed a conserved cleft that can be displaced by the binding of 2-oxoglutarate, magnesium, and ATP. Membrane fractions of Methanothermococcus thermolithotrophicus showed that GlnKs are only released in the presence of Mg-ATP and 2-oxoglutarate, leading to the structural characterization of GlnK isoforms with and without ligands (Figure 1) [9]. These results emphasize the importance of a free carboxy-terminal group for the facilitation of ligand binding and T-loop position shift in GlnKs from Methanococcales.

In this research topic series, a review article covered the topic of drug discovery.

Pang et al. reviewed the importance of aminoacyl-tRNA synthetases (aaRSs) in the translation of the genetic code, making them targets for developing anti-infective small molecules [10]. They discussed the various inhibitory mechanisms of natural and synthetic aaRS inhibitors and classified them based on their binding sites, focusing on their ability to compete with substrate association. The authors also examined the determinants of the species-selectivity and potential resistance mechanisms of some inhibitor classes. This review highlights the opportunities for further exploration of aaRSs as antimicrobial targets.

The series of articles in this publication present a comprehensive overview of cutting-edge computational and experimental studies, or a combination of both, which examine the relationship between inhibitors and target enzymes. These articles showcase the significance of the studied processes in biomedicine and the practical applications of enzyme–inhibitor interactions in pharmaceuticals, biotechnology, and other related sciences.

## Figures and Tables

**Figure 1:**
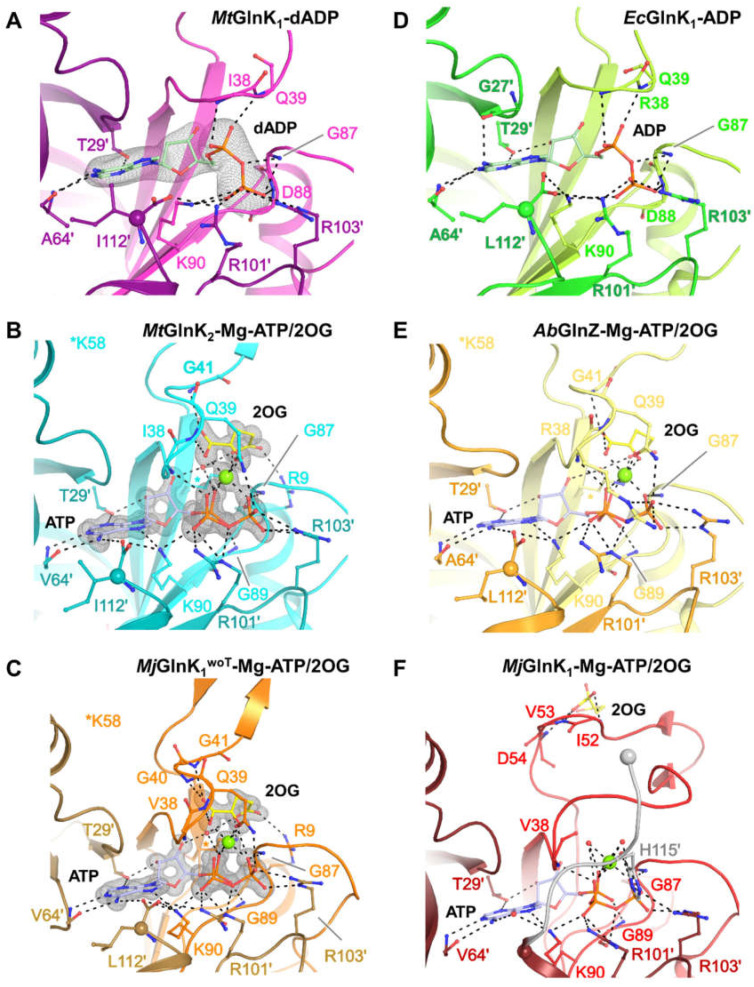
Ligand binding sites. For all panels, the main chain is represented with cartoon. Ligands and main and side chains of the residues participating in ligand binding are shown as balls and sticks. Carbon atoms are colored in yellow for 2OG, in pale green for dADP and ADP, and in pale blue for ATP, while nitrogen, oxygen, phosphorus, and Mg are colored in blue, red, orange, and green, respectively. C-termini are highlighted by a sphere. Electron density maps (2*F*_o_–*F*_c_) around the ligands are contoured at 2-σ and shown as gray mesh. Polar contacts are indicated by black dashes. (**A**) *Mt*GlnK_1_-dADP, (**B**) *Mt*GlnK_2_-Mg-ATP/2OG, (**C**) *Mj*GlnK_1_^woT^-Mg-ATP/2OG, (**D**) *E. coli* GlnK-ADP (PDB:2NUU), (**E**) *Azospirillum brasilense* GlnZ-Mg-ATP/2OG (PDB:3MHY), (**F**) tagged *Mj*GlnK_1_-Mg-ATP/2OG (PDB:2J9E) with its C-terminal tag colored in light gray. This is extracted from ref [9].
